# Clinician reports of self-awareness after traumatic brain injury: a retrospective chart review

**DOI:** 10.1186/s12913-022-08444-x

**Published:** 2022-09-06

**Authors:** Rinni Mamman, Anika Cheng, Rebecca Tsow, Julia Schmidt

**Affiliations:** 1 Graduate Program in Rehabilitation Sciences, University of British Columbia, Vancouver, Canada; 2 Rehabilitation Research Program, GF Strong Rehabilitation Centre, Vancouver, Canada; 3 Graduate Program in Occupational Therapy, University of British Columbia, Vancouver, Canada; 4 Department of Occupational Science and Occupational Therapy, University of British Columbia, Vancouver, Canada

**Keywords:** Traumatic brain injury, Self-awareness, Chart review, Occupational therapy

## Abstract

**Background:**

Impaired self-awareness (i.e., a lack of insight) is experienced by most individuals who have sustained a moderate to severe traumatic brain injury (TBI). During the early recovery period post-injury, these individuals may not be able to recognize their abilities and limitations, hence, negatively impacting their daily life and function. Although there are assessments and interventions to improve self-awareness after TBI, little is known about how clinicians assess and address this impairment in an inpatient rehabilitation setting.

**Objective:**

To examine how clinicians assess, report, and provide interventions for impaired self-awareness after TBI.

**Methods:**

A retrospective chart review was conducted on interdisciplinary rehabilitation clinician entries for individuals with TBI (*n* = 67) who received inpatient rehabilitation within a five-year period (2014–2019). A reflexive thematic analysis was used to identify themes pertaining to self-awareness.

**Results:**

Three themes were generated to explore clinician responses to their clients’ impaired self-awareness: 1) ‘recalling and understanding’ described clinician observations of client behaviors and expressions of self-awareness, 2) ‘applying and analyzing’ identified clinicians providing relevant tasks and advice to clients, and 3) ‘evaluating and creating’ described clinicians actively interacting with clients by providing feedback, guided prompts, and a follow-up plan.

**Conclusion:**

Clinicians produced varied responses to clients’ impaired self-awareness after TBI. Findings may help to develop research priorities and integrated knowledge translation initiatives to increase evidence-based practice for impaired self-awareness after TBI.

## Background

Moderate to severe traumatic brain injury (TBI) can alter a person’s physical ability, cognitive function, and behavior [1, 2]. An individual may encounter issues with balance, memory, or fatigue, causing long-term challenges that affect community re-integration and participation [3–5]. These issues often persist and can lead to life-long physical, cognitive, and psychological consequences [6]. Many individuals experience impaired self-awareness after TBI [7]. Self-awareness is conceptualised as a person’s knowledge of their abilities and limitations [8]. It is estimated that up to 97% of individuals with moderate to severe TBI have displayed impaired self-awareness, however, levels of self-awareness may improve overtime [9, 10]. The Dynamic Comprehensive Model of Awareness (DCMA) proposes self-awareness as having two components: offline awareness, the knowledge one has prior to the task (metacognitive knowledge), and online awareness, the ability to self-monitor and modify behavior during and after a task [8]. The DCMA postulates a dynamic relationship between the offline and online components, and together these concepts explain how disruptions in metacognitive knowledge and self-monitoring processes can influence an individual’s perception of their abilities when completing a task [8]. This can result in behaviors such as exhibiting poor judgement, creating unrealistic goals, and choosing to participate in activities beyond their capabilities [7, 11, 12]. Deficits in self-awareness are particularly challenging to address during rehabilitation as individuals do not recognize their limitations and therefore may not engage in therapy or use compensatory strategies [11, 13, 14]. As such, these individuals can experience difficulties in performing self-care activities, maintaining personal relationships, and sustaining a productive life [5, 15, 16].

Rehabilitation therapy for self-awareness has been identified as an essential component of multi-disciplinary rehabilitation for people with TBI [17, 18]. In order to assess self-awareness, clinicians can use various comprehensive assessments [19]. Assessing impairments in self-awareness, through standardized or non-standardized assessments, are considered a priority by clinicians and are important to promote successful outcomes [19, 20]. There are evidence-based approaches to provide interventions for self-awareness after brain injury, such as metacognitive strategy training. This can include teaching individuals to monitor their performance, identify and correct errors, and generate strategies [21] through interventions such as the use of video-based feedback [22] and pause-prompt praise [23, 24]. The multi-context approach [25] aligns closely with metacognitive training and proposes strategies that promote generalization (transferring of skills from one task to another) to enhance functional performance [26]. The key component to the multi-context approach is metacognitive training to facilitate self-awareness and self-regulation, as being able to monitor performance across different activities and contexts is crucial when generalizing skills [26]. Notably, clinical practice guidelines recognize metacognitive strategy training as a priority to address in rehabilitation [20].

Despite valid and reliable outcome measures for self-awareness and the evidence-based interventions to improve self-awareness after TBI, there is limited implementation and uptake in clinical practice [27]. One study [27] surveyed clinicians on the importance of self-awareness, and the use of instruments to assess impaired self-awareness. While this study reported a high number of clinicians who assessed self-awareness, few conducted assessments using standardized instruments specific to self-awareness. There is a gap between evidence-based recommendations and current clinical practice, in part due to clinicians experiencing barriers such as lack of time, lack of access to research, and limited research skills [28].

The objective of this study is to explore how clinicians treat self-awareness in inpatient rehabilitation settings in British Columbia, Canada. By understanding how clinicians assess and respond to impaired self-awareness in clients with TBI, we can identify how clinicians are utilizing evidence-based assessments and interventions to address self-awareness. Using a qualitative analysis, this study aims to understand the behavior and responses of clinicians when addressing self-awareness.

## Methods

### Study design

A medical record chart review was conducted. Sections relating to self-awareness were extracted from medical records of clients who received rehabilitation from GF Strong Rehabilitation Centre in British Columbia, Canada. Ethics approval was obtained by the Research Ethics Board of the University of British Columbia. The data are reported using the COnsolidated criteria for REporting Qualitative research [29].

### Data collection

A convenience sample of medical records of 67 clients were obtained from GF Strong Rehabilitation Centre, ranging from the years 2014 to 2019. Inclusion criteria for medical records were clients: 1) with a moderate to severe TBI, 2) aged over 19 years, and 3) receiving in-patient care. Authors AC and RT reviewed the medical records and extracted comments. All medical personnel were included in the analysis, regardless of discipline. Authors AC and RT examined rehabilitation goals for each client. However, clinicians did not indicate specific self-awareness goals regarding a client’s rehabilitation outcomes, but rather, reported on the client’s personal goals for recovery. Both authors extracted clinician comments about assessments, interventions, behaviors, or recommendations relating to a client’s impaired self-awareness. The clients’ demographic data, duration of stay, and cause of injury were also collected (Table 1).

**Table 1:** Client demographics from medical records

	**Clients**
Age (mean (SD))	42 (16)
Male (n (%))	50 (75)
Cause of injury (n (%):	
Motor vehicle accident (MVA)	40 (60)
Fall	10 (15)
Sports-related injuries	8 (12)
Accidents	5 (8)
Other	4 (6)
Length of stay (mean)	64 days

*Notes.* Other = Assault (1), Stab wound (1), Self-injury (1), Unknown (1)

### Reviewers

All authors of this study were female. The authors who collected data, AC and RT, were masters’ and undergraduate students, enrolled in a clinical graduate program and a bachelor’s program respectively. Both authors had previous training and experience working with people with neurological conditions. Author RM is a graduate research student while author JS is an academic researcher. All authors were situated at the University of British Columbia and have previous research experience.

### Data analysis

A reflexive thematic analysis was used to generate themes [30]. An inductive approach was applied, where data is coded without the use of a pre-existing coding guide. The following phases were used: 1) familiarization with the data, 2) generating initial codes, 3) generating themes, 4) reviewing potential themes, and 5) defining and naming themes. First, author RM reviewed researcher interpretations of the clinician comments by authors AC and RT. Second, initial codes were generated using latent coding, hence enabling researcher RM to play an active role in the interpretation of each code. Third, three themes were generated as codes were clustered together, with the themes revised overtime. Last, all themes were named to acknowledge the three different ways in which clinicians addressed their clients’ self-awareness.

The research team employed a trustworthiness strategy that involved multiple researchers in the data analysis process [31]. After each coding stage by author RM, the other authors of this paper provided input and reviewed the codes and themes, hence providing different perspectives about the data. An iterative process was applied throughout data analysis as the themes were developed.

## Results

Authors AC and RT reviewed 67 medical records. Both authors extracted 301 comments from various disciplines with the majority of comments coming from occupational therapists (Table 2). During the data collection process, authors AC and RT recorded their own interpretations of clinician comments.

**Table 2:** Number of comments from disciplines

Clinicians	n (%)
Occupational therapist	103 (34)
Nursing	56 (19)
Speech-language pathologist	43 (14)
Physical therapist	28 (9)
Medical doctor	27 (9)
Social worker	17 (6)
Physical medicine and rehabilitation	12 (4)
Recreation therapist	5 (2)
Psychology	3 (1)
Other	7 (2)

*Notes.* Other = Vocational Therapist (1), Care Management (1), Medical Representative (1), Dietician (1), Psychiatry (1), Respiratory Therapist (1), Team Rounds (1)

Three themes were identified based on the qualitative analysis of medical record entries, corresponding to clinician responses to clients’ behaviors of self-awareness: 1) recalling and understanding, 2) applying and analyzing, and 3) evaluating and creating. All clinicians, from all disciplines, reported on aspects relating to each theme. Given that there were more clinicians from the discipline of Occupational Therapy, they may have accounted for proportionally more of the recorded comments. These themes are described below with supporting quotes.

### Recalling and understanding

With this first theme, clinicians adopted an observer role in response to the client’s behaviors relating to self-awareness. Clinicians made statements about their observations of clients’ behaviors and levels of self-awareness, and at times provided descriptions of the degrees of self-awareness impairments. For example, after a session with client 15 (male, age 61) who sustained a TBI through a fall, the physical therapist noted, “limitations in rehab: decreased insight, motivation, rigid thinking”. With respect to client 17 (male, age 48) who sustained a TBI through an MVA, the physical therapist stated that the client did not co-operate during their session, “client increasingly focused on wanting to go home and not being 'forced' to do things”. Clinicians with this observer role often provided medical record entries with simple diagnoses or descriptions of the client’s level of self-awareness. For example, a speech-language pathologist observed that the client had intact self-awareness, reporting the client’s acknowledgment of speech problems and the strategies available to help. As such, they noted that client 14 (male, age 24), who sustained a TBI through an MVA, was “aware of speech difficulties and can also state strategies, however not using strategies consistently”. Within this theme, clinicians reported factually with some descriptive accounts.

### Applying and analyzing

The second theme described clinicians who moved beyond observing the issue by describing the task or activity in which the client displayed impaired self-awareness, and the subsequent advice they provided the client. A speech-language pathologist of client 7 (female, age 61) who sustained a TBI due to a fall, indicated that despite displaying cognitive-communication impairments during a task, the client was unconcerned about these difficulties. The client’s vocational therapist stated their attempts to mitigate these challenges by offering “a draft alternate return-to-work plan with a longer timeframe than the planned 3-week schedule”. However, the client declined this as she did not feel she required more time before returning to her vocational role. Clinician responses within this theme also included descriptions of the context of the self-awareness behaviors. The physician of client 5 (male, age 48) who sustained his TBI through an MVA, expressed concern with the client’s sugar levels:

Client stated he has been doing own sugar adjustment for years, can do it himself. Writer reviewed history of TBI, determined client may be at some risk but client feels able to manage at home safely.

In a different session, the nurse observed the client was unable to generate a reading using a glucometer. The nurse reported the “client subsequently refused to make a second attempt despite multiple requests to demonstrate competence with this tool”. In both instances, the clinicians reported that the client presented with avoidance and denial, potentially as coping strategies, in their task performances and described their advice to the client regarding their behavior of impaired self-awareness.

### Evaluating and creating

Clinicians actively engaged with the client within this third theme, explicitly outlining a comprehensive description about the session, the client’s portrayal of their level of self-awareness, and a follow-up plan. The psychologist of client 64 (female, age 28) who sustained a TBI through an MVA, indicated the abilities they observed and their plan to incorporate resources for the client outside the rehabilitation centre:

Sessions have focused primarily on processing emotional responses to post-injury changes in functioning, relationships, and sense of self. She shows excellent insight into her affective experience and engaged well in sessions. Writer has assisted client in further developing awareness of her emotional responses …writer will follow up with client regarding available mental health resources in her home community and nearby.

Another session with a speech-language pathologist of client 66 (male, age 21), who sustained a TBI due to a stab wound, noted the areas that the client experienced difficulties. They assessed the client’s self-awareness regarding these deficits, incorporated their reasons behind their evaluation, and described a plan to consult other clinicians:

Most noted difficulties with visual memory, new learning and prospective memory and spatial memory…Client noted to overestimate abilities/performance pre- and post- assessment…Discussed strategies of repetition, talking aloud, and making notes. Liaised with speech-language pathologist and medical doctor about client's fatigue and low sustained attention in sessions. Plan- focus on assessment of home and community tasks next week.

As described in this theme, the clinicians reported their active engagement with the client, the behavior of the client that helped formulate the advice given, and the next steps needed to help the client.

## Discussion

This study identified three themes that explore how clinicians address self-awareness in a rehabilitation setting: ‘recalling and understanding’, ‘applying and analyzing’, and ‘evaluating and creating’. All three themes of clinician behaviors indicate the key role of conducting assessments and providing interventions for self-awareness after TBI. Findings provide insight to the varying types of responses and differing degrees of understanding of evidence and implementation.

The importance of observation was evident in the theme of ‘recalling and understanding’. By observing a client’s task performance, clinicians target a client’s online awareness that is activated during a performance [32]. This is essential as to choose the best treatment plan, clinicians need to recall relevant knowledge and understand whether clients can self-monitor and adjust their behavior accordingly. However, using this form of assessment is not common as an online survey identified only 5.5% clinicians who assess self-awareness after a brain injury by using unstructured observations [27].

With the second theme ‘applying and analyzing’, clinicians moved beyond simply describing the behavior or stating the client’s self-awareness by providing a descriptive account about their session and reporting on the task and advice delivered. Clinicians facilitated occupation-based sessions aligned to client-based goals (e.g., driving skills, cooking tasks, work-oriented activities). Clinician reports within this theme align to research indicating that selecting tasks that correspond with the clients’ personal goals help to improve participation and motivation during rehabilitation resulting in improvements in self-awareness [33, 34]. The clinician comments indicate an attempt to identify barriers that the client faced and offer task-specific advice and direction. However, research suggests that employing occupation-based metacognitive strategy training may be more effective than directing clients to adjust their behavior, as administered by the clinicians with this theme [35]. Additionally, the use of techniques such as self-monitoring, self-correcting, and self-evaluating may guide the client to clearly associate this strategy with functional gains [36]. This aligns with the DCMA, as this model offers an occupation-based explanation for self-awareness, hence improving a person’s awareness during a performance can result in improvements in their metacognitive knowledge [8].

With the third theme, ‘evaluating and creating’, clinicians actively interacted with clients by providing verbal feedback, encouraging use of strategies, and exploring external resources to improve self-awareness. Clinicians primarily used verbal feedback within an occupation-based approach. Research suggests that additional forms of feedback (e.g., video-based feedback) can add to the efficacy of feedback interventions [22]. This theme was most aligned with clinical practice guidelines on cognitive rehabilitation as all the guidelines reviewed suggested behavioral interventions, the use of feedback, and group therapy to address self-awareness and insight [37].

There were limited reports of self-awareness assessment use with clinicians reporting on clients' levels of self-awareness, task-specific challenges, and interventions for self-awareness. Research has shown that self-awareness assessments are important in order to assess and evaluate different components of a client’s self-awareness [38]. Additionally, components such as anticipatory awareness needs to be objectively assessed by a clinician [38]. In our study, less than 10% of clients were reported as receiving a self-awareness assessment. This aligns with the findings of an online survey study, as 77% of clinicians reported not using instruments to assess self-awareness after a brain injury [27]. This may be due to factors such as time constraints [39, 40] or lack of knowledge. The time needed to administer a self-awareness test depends on the participant, as clients who have a higher level of cognitive fatigue may need a longer period of time to complete the assessment. Additionally, clinicians may not have required the training to address self-awareness during rehabilitation. A survey study indicated that self-awareness was identified by clinicians as the one of the top three preferences for professional development and a leading barrier in providing successful rehabilitation to clients [17].

Research indicates that TBI rehabilitation is most effective if delivered through a holistic, multidisciplinary team [17, 41]. While our study does not address the outcomes of delivering holistic treatments, our study identified 17 different clinician disciplines that interacted with clients who experienced changes to their self-awareness. This interdisciplinary rehabilitation structure is essential to assess and treat impaired self-awareness, as it affects all aspects of an individual’s rehabilitation progress, ranging from cognitive issues to their physical abilities [42]. The biopsychosocial model of awareness indicates there are interacting factors at biological, psychological, and social levels that can influence individuals’ presentation of self-awareness deficits [43]. For example, individuals may display denial, minimization, or avoidant coping strategies, which are the result of defensive mechanisms at the psychological level. These individuals may benefit most from psychologists or counsellors to understand the meaning of the impairments and denial reactions [44]. Alternatively, an individual that displays impaired self-awareness on community re-integration tasks (e.g., crossing the road) may benefit from occupational therapy intervention to facilitate metacognitive strategy training within an occupation-based framework. As a result, having an interdisciplinary framework emphasizes the idea of a holistic need in rehabilitation.

### Relating to theory

These themes are situated within the revised version of Bloom’s Taxonomy [45], which emphasizes the importance of creating rather than synthesizing, higher levels of cognitive skills, and the shift towards more dynamic classifications. The revised taxonomy proposes a progressive cognitive hierarchy that encompasses six levels: remember, understand, apply, analyze, evaluate, and create. Utilizing this encourages deeper learning and the generalization of skills and knowledge to a variety of tasks and contexts [46]. The themes are framed and described within this learning theory as health professionals, who aim to develop and achieve high level skills and function, require deeper cognitive processing including critical thinking and judgement [46]. This can explain the level of learning, implementation of learning, and provide evidence for knowledge translation at each stage of clinician behavior.

The findings represent three different ways that clinicians reported observations and interventions related to self-awareness that they encountered in their practice. It shows that a lack of self-awareness and insight is recognised by an interdisciplinary team of clinicians and is addressed by each clinician. The first theme describes the observant role of a clinician in which general comments about the client’s self-awareness are made. This aligns with the concept of ‘remember and understand’, where clinicians recognize, describe, and summarize what is being observed. This is considered a foundational cognitive skill that represents the beginning stages of learning behavior. The second theme explores clinician comments that move beyond the general identification of self-awareness and include descriptions of the tasks the client performed, and the advice provided. This theme is described by the concept ‘apply and analyze’, which utilizes the clinician’s skills to mitigate challenges, and to further explore and infer the reasoning behind the presentations of impaired self-awareness. Finally, the third theme represents clinicians who actively engaged with clients, providing detailed descriptions of the sessions as well as a follow-up plan. At this stage, clinicians are achieving higher levels of critical thinking and judgement through evaluation and recommendations to create a plan of action for their client.

### Limitations

There are three limitations in this study. First, clinicians have different styles of reporting client sessions, and thus, may choose not to report on certain aspects of the session, such as self-awareness behaviours. Clinicians may have been less descriptive when documenting, due to factors such as time constraints, which may not reflect their behavior and responses in the session itself. There may also have been clinicians who recorded their sessions in a manner that depicted their treatment plans and behavior in a favourable light. Hence, clinicians’ reports may have described that the clients’ lack of progress may be caused by the client themselves, as opposed to the skills of the therapist. In addition, changes in clinicians due to external factors (i.e., vacation, sick days, etc.) may result in missed reports of impaired self-awareness in the medical records as some clients may rely on compensatory strategies to conceal their deficits. However, these findings can provide key information for future researchers to develop a longitudinal observational study of clinician behavior when engaging with clients with impaired self-awareness. Second, no demographic information could be obtained about the clinicians in our study (e.g., age, clinical experience, years working with individuals with TBI) due to the nature of the retrospective chart review design. Some of these factors may have influenced how clinicians document their sessions. Third, the limited sample size and examination of records from only one rehabilitation centre may not reflect the findings that could be obtained from other sites. The in-service training provided to the clinicians in this rehabilitation centre may differ from other centres.

## Conclusion

This study identified three themes using a reflexive thematic analysis that illustrate clinicians’ responses to self-awareness behaviors within an inpatient rehabilitation setting. Findings may help to facilitate future research priorities on the use of evidence-based approach to self-awareness management after TBI and may develop implementation and knowledge translation activities for self-awareness assessments and interventions in rehabilitation settings.

## Data Availability

Data sharing is not applicable to this article as no datasets were generated or analysed during the current study.

## Abbreviations

DCMA: Dynamic Comprehensive Model of Awareness
MVA: Motor vehicle accident
TBI: Traumatic brain injury
